# Survival outcomes in patients with sigmoid volvulus

**DOI:** 10.1007/s00384-025-04920-y

**Published:** 2025-06-17

**Authors:** Rosa D. E. Bock, Peter G. Vaughan-Shaw, Edinburgh Colorectal Group, A. J. Clark, M. Collie, D. Collins, M. Duff, S. Goodbrand, J. Mander, N. T. Ventham, H. M. Paterson, M. A. Potter, C. Reddy, D. Speake, F. V. N. Din, M. G. Dunlop, G. Smith

**Affiliations:** Western General Hospital, Edinburgh, EH42XU UK

**Keywords:** Sigmoid volvulus, Elderly, Frailty, Surgery

## Abstract

**Aim:**

This study aimed to assess management pathways and outcomes in sigmoid volvulus (SV).

**Methods:**

A retrospective review was performed on patients first admitted with SV between 2019 and 2023 within a tertiary-level colorectal service. Demographic, management, and outcome data, including frailty, ASA (American Society of Anaesthesiologists), and National Emergency Laparotomy Audit (NELA) score, were collected. Comparative statistics were used to compare baseline demographics between those operated on and those not and to identify factors associated with survival.

**Results:**

A total of 72 patients were included, median age of 78 years, with 25 undergoing surgery. After index discharge without surgery, 50 patients (88%) were re-admitted with SV at least once, with a total of 212 hospital admissions and 1952 hospital bed days at the end of follow-up. A trend towards lower age, NELA score, ASA score and frailty score was seen in those undergoing surgery, with only two deaths observed during postoperative follow-up. In those who were not palliated at first admission but did not undergo surgery at any point, the mortality rate was 42% (*n* = 16, median survival 545 days, median age 79), with causes of death generally reflecting conditions of frailty and not volvulus itself.

**Conclusions:**

This study demonstrates the burden of sigmoid volvulus in an elderly population with significant mortality and morbidity. While survival was better in those undergoing surgery, this likely represents appropriate case selection reflecting underlying frailty and comorbidities in those not offered surgery rather than a protective effect of surgery. While surgery should be considered and documented at index admission, it should not be considered a panacea for the elderly and frail population.

**What does this paper add to the literature?:**

This study highlights the burden of sigmoid volvulus in an ageing population, emphasising complex management challenges. Non-operative treatments showed high recurrence and poor survival, while surgery yielded excellent outcomes in selected patients. The findings advocate for a cautious, individualised approach, balancing frailty and risks, rather than universal reliance on surgery.

**Supplementary Information:**

The online version contains supplementary material available at 10.1007/s00384-025-04920-y.

## Introduction

Sigmoid volvulus (SV) is characterised by torsion of the sigmoid colon and is a common cause of large bowel obstruction, predominantly affecting elderly patients [1]. The elderly are at greater risk due to increased weakening and loss of elasticity of the colonic musculature, increased constipation and predisposing comorbidities, such as chronic neurological disorders [1–3].

Acute management of sigmoid volvulus is generally by rectal tube or sigmoidoscopic decompression unless ischaemia or perforation indicates urgent surgery. Recent guidelines from the World Society of Emergency Surgery (WSES) recommend that elective sigmoid colectomy is considered early after the first presentation and even during the index admission [4]. The recommendation is reported as ‘strong’ yet is based on low-quality evidence. The recommendation is justified by high recurrence rates of 45–71% following endoscopic decompression and low morbidity and mortality following non-urgent sigmoid resection [4]. However, the patient cohorts from which it is drawn have an average age of ~ 58–68, as reported in the key cited papers. This is markedly lower than the age anecdotally observed in our current surgical practice [5–8] and may limit the generalizability of these recommendations to current surgical patients.

The reported management of SV in some centres is endoscopic decompression alone, with elective surgical intervention relatively uncommon [9]. The argument for elective surgery is based on high rates of recurrence and the morbidity and mortality from the cycle of recurrence, hospital readmission and patient deconditioning [1, 2, 10], with patients often suffering multiple recurrences throughout their lifetime [4, 10]. The cumulative effect on the individual and healthcare system is significant, while each recurrence risks ischaemia and perforation [3, 11]. Surgery may have a role in reducing this burden but has significant risks in an elderly, frail and deconditioned patient.

This study aimed to define management pathways in patients diagnosed with SV and to compare the recurrence and mortality among patients managed with a non-operative or operative approach.

## Methods

A retrospective review was performed on patients admitted with SV within NHS Lothian, a tertiary-level colorectal service in South-East Scotland. Approval was granted by the local Caldicott Guardian and Quality Improvement Lead.

Patients admitted with SV between January 2019 and December 2023 were identified through a free text search of radiology reports. Electronic medical records were interrogated to confirm eligibility. Patients were excluded if they had previous admissions with confirmed SV before January 2019. Demographic, management and outcome data were extracted from electronic medical records for the index and subsequent admissions, including Rockwood Clinical Frailty score, ASA (American Society of Anaesthesiologists) grade, NELA (National Emergency Laparotomy Audit) score and Clavien-Dindo complication grade [12, 13] for all patients. Summary data for number of admissions has been rounded to whole numbers. Follow-up data was collected from patient admission to June 2024. All-cause mortality was collected until June 2024. Selection and follow-up data included all regional hospitals, ensuring a comprehensive regional dataset.

Data were collated in Microsoft Excel [14]. For quantitative variables, non-parametric data are presented as median and interquartile range (IQR), while NELA score is presented as mean with a 95% confidence interval (CI). The Mann–Whitney *U* test was used to test the association between variables, and the chi-squared test was used to test the proportions of NELA scores < 5%. Univariate survival analysis was performed using the Kaplan–Meier (K-M) methodology, and the significance of differences between survival curves was determined by the log-rank test. Cox regression modelling was used to identify factors associated with survival in patients with sigmoid volvulus. *P* < 0.05 was considered statistically significant for all tests, accepting the small risk of a type 1 error due to multiple comparisons. The study adhered to the STROBE reporting recommendation [15].

## Results

**Fig. 1:**
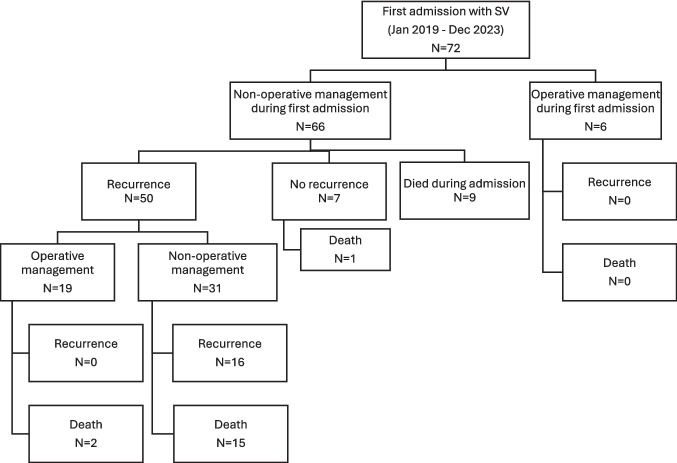
Overview of patient pathways

A total of 72 patients were included, with a median age of 78 (range 43–95), with 72% being over 70 years old. Demographics at index admission are shown in Table 1. Forty-seven patients were managed non-operatively across all admissions, while 25 underwent surgery (Fig. 1).

**Table 1:** Patient demographics and commonly used operative scoring

Variables	Non-operative (*n* = 47)	Operative (*n* = 25)	*P* value
Gender (Female)	17 (36%)	9 (36%)	0.99
Age (IQR)	80 (72–85)	73 (64.5–84.5)	0.1
ASA grade	3 (2–3)	2 (2–3)	0.22
Frailty score	6 (4–6)	4 (3–6)	0.67
NELA score (%) (95% CI)	5.32 (4.12–6.56)	4.68 (3.12–6.24)	0.64
NELA score > 5 (%)	16 (34%)	7 (28%)	0.60

In the operative group, 5 elective procedures and 20 emergency or expedited procedures were performed, with 13 (52%) having an end colostomy and 3 (12%) requiring a total colectomy and ileostomy. When comparing those who had surgery and those who did not, age, NELA score and ASA were higher in the non-operative group, although these trends did not reach statistical significance [12]. Those in the non-operative group appeared to be frailer, albeit without statistical significance, with a median clinical frailty score of ‘moderately frail’, compared to the lower category of ‘vulnerable’ in those undergoing surgery [16].

Nine patients died during the index admission, including from colonic perforation (*n* = 2), bowel obstruction (*n* = 2), pneumonia (*n* = 3), cardiac arrest (*n* = 1) and cardiac failure (*n* = 1); these patients were treated palliatively and were not considered candidates for resectional surgery from the outset. In the operative group, there were no deaths at 90 days, with four patients (16%) suffering a Clavien-Dindo grade 3 or 4 complication: One patient required a negative relook laparotomy due to suspected postoperative bleeding. Three patients required admission to ICU for intubation and/or the use of vasopressors, including one patient following re-operation for anastomotic leak (Supplementary Table 1). No patient died within 90 days of surgery.

The included patients (*n* = 72) accounted for a total of 212 hospital admissions for SV and 1952 hospital bed days at the end of follow-up. One patient had 10 admissions over the 4-year study period with a total hospital stay of 95 days. Of the 57 patients discharged alive and without surgery following the index admission, 50 (88%) were re-admitted with SV at least once (Table 2). Patients undergoing surgery had more prior admissions and more decompressions before surgery than those in the non-operative group; of those 19 patients undergoing surgery after recurrent SV, 11 (58%) had had ≥ 4 admissions, compared to 10 (26%) patients in the non-operative group (*P* = 0.04). Meanwhile, 9 (47%) patients who ultimately received surgery had ≥ 4 endoscopic decompressions compared to 14 (37%) in the non-operative group (*P* > 0.05; Supplementary Table 2). In a subset of 24 patients first admitted with SV in 2023, clinical case note analysis identified a documented contra-indication or discussion regarding surgical management in only seven patients (29%) in the non-operative group.

**Table 2:** Median number of admissions with SV, endoscopic decompressions and hospital days per patient (IQR) in non-operative and operative groups

Variable	Non-operative	Operative
Number of admissions with SV	2 (1–3)	3 (2–6)
Number of endoscopic decompressions	2 (1–4)	3 (1–6)
Cumulative number of hospital days per patient	14 (8.8–26.8)	23 (15–33.5)

Median follow-up was 584.5 days (range 2–1903). At the end of the follow-up, the overall mortality rate was 8% (*n* = 2) in those undergoing surgery (*n* = 1 cardiac failure, *n* = 1 stroke). In those who were not palliated at first admission and did not undergo surgery at any point, the mortality rate was 42% (*n* = 16, median survival 426 days from index admission, median age 83), with 10 deaths occurring in the community (cause unknown). Of those who died during hospital readmission (*n* = 6), the cause of death reflected general frailty (*n* = 2 urosepsis, *n* = 2 fall, *n* = 1 overdose, *n* = 1 pneumonia).

**Fig. 2:**
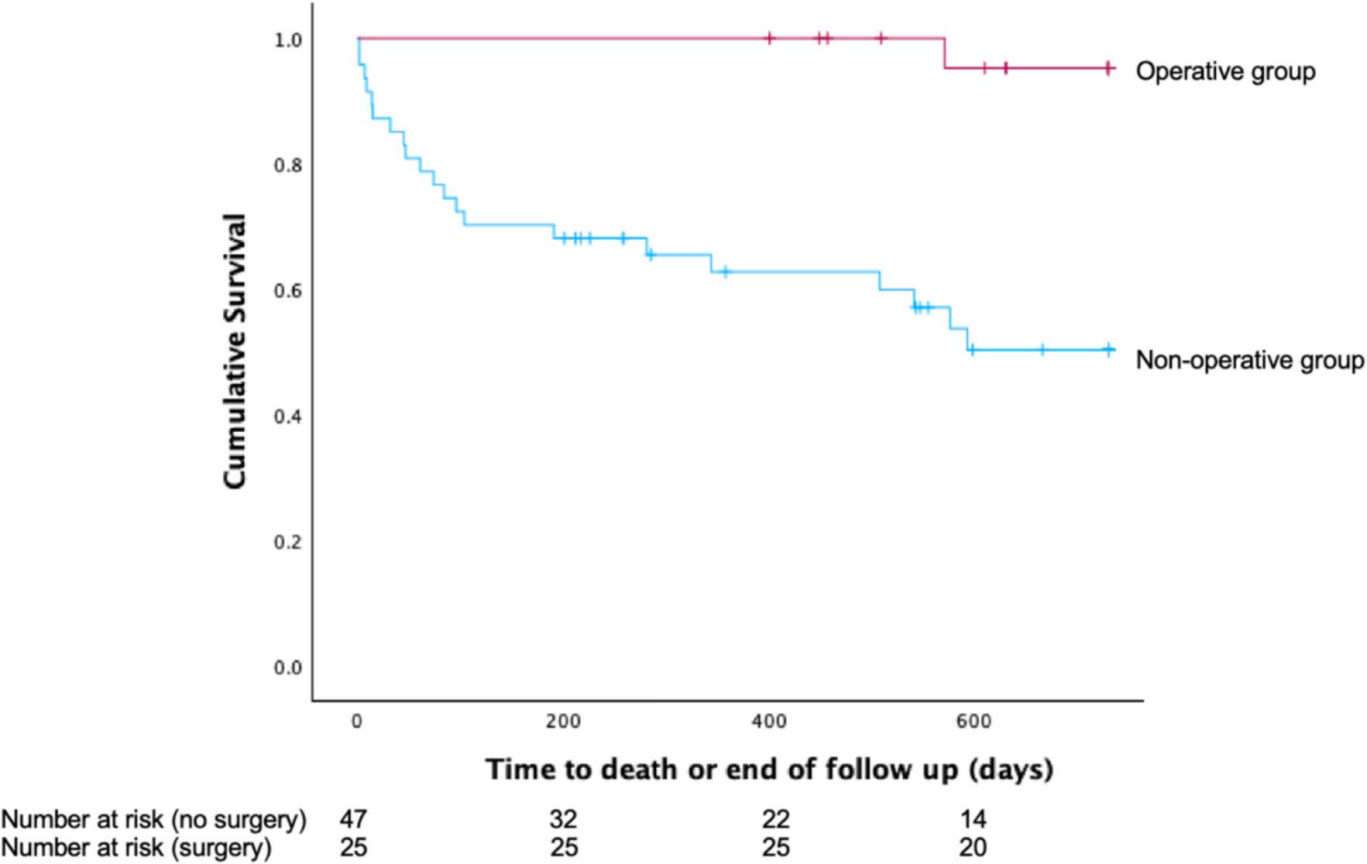
Kaplan–Meier 2-year survival curve comparing non-operative and operative groups

On survival analysis, lower age (HR 1.06, 95% CI 1.00–1.13) and operative intervention (HR 0.07, 95% CI 0.01–0.54) were associated with greater 2-year survival (Fig. 2; Supplementary Table 3), yet NELA score, sex and frailty score were not. Patient age and surgery remained associated with survival after removing non-significant variables (Supplementary Tables 4 and 5) when excluding those patients who were palliated at first admission and the end of follow-up.

## Discussion

This study demonstrates a significant burden of sigmoid volvulus in an elderly population with significant mortality and morbidity. This burden, both at a personal and healthcare level, is expected to increase with an increasingly elderly and frail population [17].

Following index diagnosis, all patients in our study were initially managed with endoscopic decompression, an effective initial management strategy due to its minimally invasive nature and high success rate [4, 17], avoiding general anaesthesia and its associated risks [18, 19]. However, 88% of patients who did not undergo surgery but survived the index admission were re-admitted with recurrent volvulus. Many patients had multiple recurrences, resulting in repeated admissions, decompressions and, in some, eventual surgery. The burden of numerous recurrences on the individual and healthcare system is demonstrated by the large number of endoscopic decompressions (generally done in the operating theatre) and hospital bed days each patient accumulated. High rates of recurrence are confirmed in the published literature [4, 9, 20–22]: Moro-Valdezate reported 47.2% of patients experienced SV recurrence after endoscopic decompression, while [21] Johansson et al. reported 84% recurrence in a Swedish cohort [9]. With each future episode of sigmoid volvulus, there are cumulative risks of deconditioning and even acute ischaemia or perforation, which is itself accompanied by a significant mortality risk [4].

Surgery significantly reduces the risk of future recurrence of SV [4, 9, 21, 23]. Recognising this, the WSES guidelines recommend early consideration of sigmoid colectomy, ideally at or soon after index admission, to prevent recurrent SV [4]. Only ~ 35% of SV patients in our study received surgery following endoscopic decompression, while similar studies report operative rates of 35–63% [2, 9, 10, 22]. However, these studies and those cited by the WSES guidelines have markedly lower patient median/mean age than the current study, limiting direct comparability. Patients in our study also often had several admissions with SV before surgery was performed, suggesting they could have undergone surgery at an earlier stage. Given the observed high risk of recurrence, it is clear that robust, proactive decision-making should be completed and documented at an earlier opportunity. Certainly, as Johansson et al. suggest, a second presentation (i.e. recurrent SV) should prompt such discussions [9]. The low rate of documentation of assessment for surgery in our subset analysis should act as a quality improvement target and prompt education around SV pathways.

In our cohort, postoperative outcomes were excellent, with very low mortality observed among patients undergoing surgery — consistent with similar studies reporting mortality rates of 5.4–7% [9, 10] — suggesting effective case selection. In contrast, survival was poor among those who did not receive surgery, although causes of death were generally unrelated to SV itself and instead reflected broader frailty-related conditions such as pneumonia or falls.

Surgical decision-making in this situation is complex. Most patients in this cohort were elderly with varying degrees of pre-existing frailty, rendering major surgery inappropriate for some. On the other hand, conservative management exposes patients to recurrent episodes of SV and repeated hospital admissions, which themselves are recognised contributors to deconditioning and loss of functional independence in elderly patients [18, 24]. At our centre, a care of the elderly team is involved in the assessment of some surgical patients, and their input is invaluable in guiding these complex decisions. A comprehensive geriatric assessment during admission, including frailty scoring, functional baseline evaluation and care escalation planning, should provide invaluable insight into surgical fitness and inform shared, patient-centred decision-making [24].

While this study captured recurrence rates and survival benefits associated with surgical intervention for SV, important metrics such as changes in mobility, discharge destination, care dependency and stoma-related quality of life were not assessed. These outcomes are crucial when considering the broader impact of surgery in frail patients, balancing recurrence prevention and preservation of function and independence [24].

There is no one-size-fits-all approach to managing SV in this population, and no study to date has definitively demonstrated the survival benefit of surgery. Future research must assess the impact of surgery on frailty progression, functional status and long-term quality of life. These insights would better inform treatment decisions and help guide when surgical intervention is beneficial. We encourage research funders to prioritise outcome setting and robust collaborative multicentre studies into SV.

Advanced endoscopic techniques, such as percutaneous endoscopic colostomy, have previously been described as a less invasive technique to prevent SV recurrence in high-risk surgical patients [4, 10] but were not performed in patients in the current dataset. Indeed, while the WSES guidelines offer only weak recommendations for their use based on low-quality evidence, the absence of this intervention in our series is notable [4]. Given the frailty of our cohort and high recurrence rates, this technique has the potential to be beneficial, and further prospective evaluation of it may be warranted.

This study had several limitations, primarily due to its retrospective nature and small sample size. Only a randomised trial or a well-designed propensity-matched study could define whether surgery improves survival; otherwise, selection bias is a critical confounder in any retrospective analyses. We acknowledge that patients in the non-operative group who died in the community may have died from complications relating to the recurrence of SV. Furthermore, we acknowledge that without protocolised follow-up, patients may have moved away from the area impacting the capture of recurrence data, although given that our clinical data capture covered a large tertiary area and the age of this cohort, we consider this relatively unlikely.

Despite the small size, the granular data from a smaller cohort have allowed a more focused analysis of each patient pathway and a comparison of current practice to published WSES guidelines. A non-significant trend in lower age, frailty and NELA score suggests that those who received surgery were generally younger and fitter, indicating clear selection bias in survival comparisons. Meanwhile, other relevant demographics, e.g., detailed comorbidity scoring and nursing/care home residence, were not collected and would inform surgical decision-making. The findings from this study are likely broadly relevant to other healthcare settings with ageing populations and similar burdens of frailty. However, generalisability may be limited in countries with differing healthcare access, infrastructure or thresholds for surgical intervention. Future multicentre or international studies would help to validate these findings across diverse healthcare systems. Additionally, further research looking at early elective surgery, percutaneous endoscopic colostomy and functional outcomes would provide better insight into the management of SV in elderly patients.

This study describes the significant burden of sigmoid volvulus in a single centre and outlines the current management pathways with reference to recent guidelines. The population is unsurprisingly elderly and frail, with a higher median age than many similar studies, including those that informed the recent WSES guidelines. It is clear that an early objective assessment of fitness for surgery is essential to aid shared decision-making and inform the role of surgery in preventing multiple readmissions, with the associated risks and burden to both the individual and the healthcare system. However, given the significant frailty and older age and poor observed survival in those patients, surgery is unlikely to extend life significantly and should not be undertaken without appropriate discussion and multi-disciplinary team input.

## Supplementary Information

Below is the link to the electronic supplementary material.

Supplementary file1 (DOCX 34 KB)

Supplementary file2 (DOCX 18 KB)

## Data Availability

Data is provided within the manuscript or supplementary information files. Anonymised data and statistical code available on request.

## Abbreviations

ASA: American Society of Anaesthesiologists
CI: Confidence interval
HR: Hazard ratio
IQR: Interquartile range
K-M: Kaplan-Meier
NELA: National Emergency Laparotomy Audit
SV: Sigmoid volvulus
WSES: World Society of Emergency Surgery
