# Supplementary material for: Survival outcomes in patients with sigmoid volvulus
Source: Int J Colorectal Dis. 2025 Jun 17;40(1):142. doi: 10.1007/s00384-025-04920-y (PMC12174257; doi:10.1007/s00384-025-04920-y)
Supplement: Supplementary file 2 — Supplementary file2 (DOCX 18 KB) [file 384_2025_4920_MOESM2_ESM.docx]

**Failure to consider surgical intervention risks recurrence and adverse outcomes in elderly patients with sigmoid volvulus**

RDE Bock^1^, PG Vaughan-Shaw^1^, Edinburgh Colorectal Group

**Supplementary data**

Supplementary Table 1.

| Clavien-Dindo grade | Non-operative | Operative |
| --- | --- | --- |
| 0 | 25 | 12 |
| 1 | 13 | 8 |
| 2 | 0 | 1 |
| 3 | 0 | 2 |
| 4 | 0 | 2 |
| 5 | 9 | 0 |

Clavien-Dindo complication grades.

Supplementary Table 2.

| Variable | Number | Non-operative | Operative |
| --- | --- | --- | --- |
| Total number of admissions with SV | 1 | 16 | 6 |
|  | 2 | 15 | 5 |
|  | 3 | 6 | 3 |
|  | ≥4 | 10 | 11 |
| Total number of endoscopic decompressions | 1 | 11 | 7 |
|  | 2 | 12 | 5 |
|  | 3 | 7 | 4 |
|  | ≥4 | 14 | 9 |

Number of admissions with SV, total number of hospital days and number of endoscopic decompressions in non-operative and operative groups.

Supplementary Table 3.

|  | HR | 95% CI | P value |
| --- | --- | --- | --- |
| Surgery (Yes) | 0.07 | 0.01-0.54 | 0.010 |
| Age | 1.06 | 1.00-1.13 | 0.046 |
| NELA | 634.24 | 0.03-NA | 0.197 |
| Sex (Male) | 1.96 | 0.71-5.46 | 0.196 |
| Frailty | 1.00 | 0.72 | 0.995 |

Cox regression analysis for 2-year survival

Supplementary Table 4.

|  | HR | 95%CI | 95%CI | P |
| --- | --- | --- | --- | --- |
| Surgery (yes) | 0.07 | 0.01 | 0.54 | 0.01 |
| Age | 1.06 | 1.00 | 1.12 | 0.04 |

Cox regression analysis for 2 year survival after removing NELA score, sex and frailty

Supplementary Table 5.

|  | HR | 95%CI | 95%CI | P |
| --- | --- | --- | --- | --- |
| Surgery (yes) | 0.12 | 0.03 | 0.50 | 0.004 |
| Age | 1.07 | 1.01 | 1.12 | 0.01 |

Cox regression analysis for overall survival after removing NELA score, sex and frailty
